# Template-based landmark and region mapping of bone

**DOI:** 10.1186/1757-1146-7-S1-A42

**Published:** 2014-04-08

**Authors:** Jaeil Kim, Sang Gyo Seo, Dong Yeon Lee, Jinah Park

**Affiliations:** 1 Department of Computer Science, Korea Advanced Institute of Science and Technology, Daejeon, South Korea; 2 Orthopedic Surgery, Seoul National University Hospital, Seoul, South Korea

## Background

The shape morphology using 3D surface models has been recently emerged for biomechanics research, such as the quantitative assessment of bone deformity with clinical factors [1] and the correlation analysis between bone shape and joint motion [2]. In the bone shape morphology, the morphological difference of the bones across subjects is quantified by the geometric measures, such as the curvature of the articular surface and the relative bone orientation in joints, defined with the anatomical landmarks and regions on the bone surface. However, the landmark and region determination on individual cases is a difficult and time-consuming task, because of the various size and shape of the bones and operator’s errors.

In this paper, we propose an automated landmark and region mapping method based on a non-rigid template-to-image registration. The template model is a triangular mesh including the generic shape of the target. It also encodes the landmarks and regions as a subset of the points in the triangular mesh. For the landmark and region mapping to individual bones, the template model is non-rigidly deformed by a Laplacian deformation framework [3]. This framework derives the point transformation into the image boundary while minimizing the distortion of the point distribution in the template model. This behavior of the deformation framework helps to trace the positions of the anatomical landmarks and regions across subjects.

## Results

**Figure 1:**
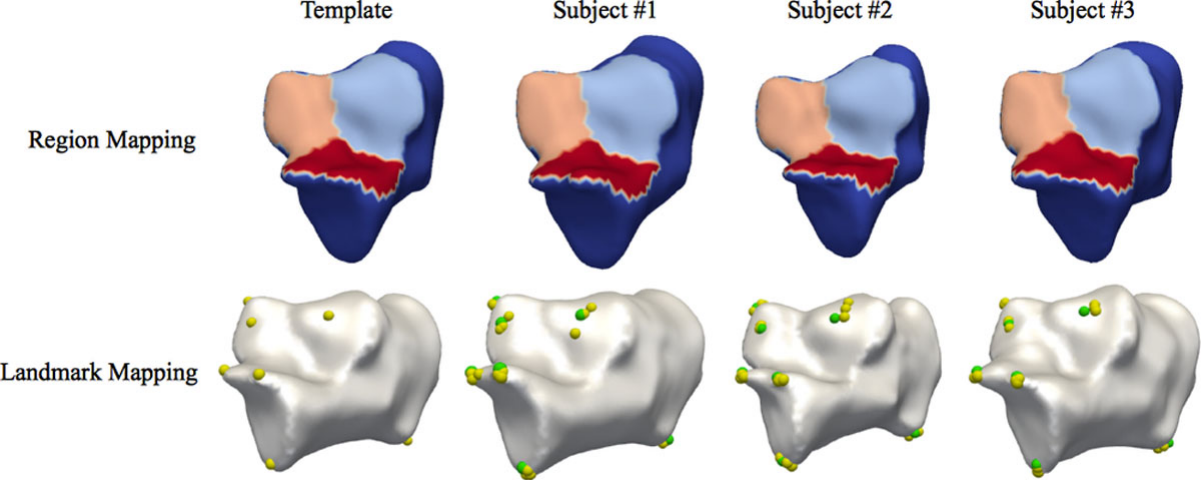
Articular surface and landmarks, which are automatically identified for the calcanei of 3 subjects

For our experiment, the calcaneus template model was constructed from the manually segmented CT scans. We assigned 7 landmarks and the articular surface for the talus in the calcaneus model. We applied our method to the segmentations of three subjects having the different size calcanei. Figure 1 (top) shows the reconstructed models with the articular surfaces, which were automatically labeled, shown in different colors. The accuracy of the individual shape reconstruction was a volume overlap (complete overlap=1.0) of 0.981±0.009 and a mean distance of 0.349±0.423 mm with respect to the segmentations. To validate the consistency of the automatic landmark determination, three blinded operators manually assigned the landmarks in the reconstructed models. Considering the inter-operator variations, the automatic landmarks (green) were consistent with the manual landmarks (yellow) in the template and individual models, as shows in Figure 1 (bottom). These results indicate that our method determines the accurate positions of the anatomical landmarks and regions while restoring the individual shape characteristics of bones. We plan to assess the robustness and accuracy of the automatic landmark and region mapping with larger datasets.
